# Robot-assisted resection of a dumbbell-shaped intradural tumor in the prone position

**DOI:** 10.1016/j.xjtc.2022.06.015

**Published:** 2022-07-02

**Authors:** Yoshimitsu Hirai, Hiroshi Iwasaki, Hideto Iguchi, Aya Fusamoto, Yumi Yata

**Affiliations:** a Department of Thoracic and Cardiovascular Surgery, Wakayama Medical University, Wakayama, Japan; b Department of Orthopedic Surgery, Wakayama Medical University, Wakayama, Japan

**Figure:**
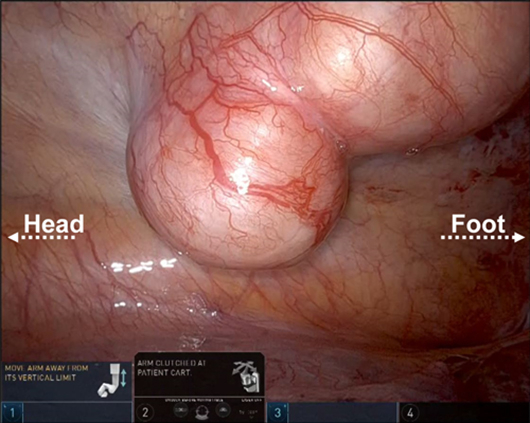
Intraoperative view of the tumor.

**Central Message**

Robot-assisted approach of a dumbbell-shaped intradural tumor in the prone position is useful and presents many practical advantages.

The resection of Eden type 2 or 3 dumbbell-shaped tumors has traditionally been performed by a combination of prone laminectomy and lateral video-assisted thoracic surgery (VATS).^1^ Okazaki and colleagues^2^ reported the usefulness of robot-assisted intrathoracic procedure for the resection of epidural dumbbell-shaped dorsal spine tumors, with the patient in the prone position and without need for repositioning. Here, we were performed a robot-assisted thoracoscopic surgery (RATS) for an Eden type 2 schwannoma with intradural extension. In this article, we report on the various innovations we have made to resect dumbbell-shaped tumor extending into the dura mater using a posterior approach and RATS, with the patient in the prone position and without repositioning. Institutional review board approval was not required, and the patient provided informed written consent.

## Case Presentation

**Figure 1:**
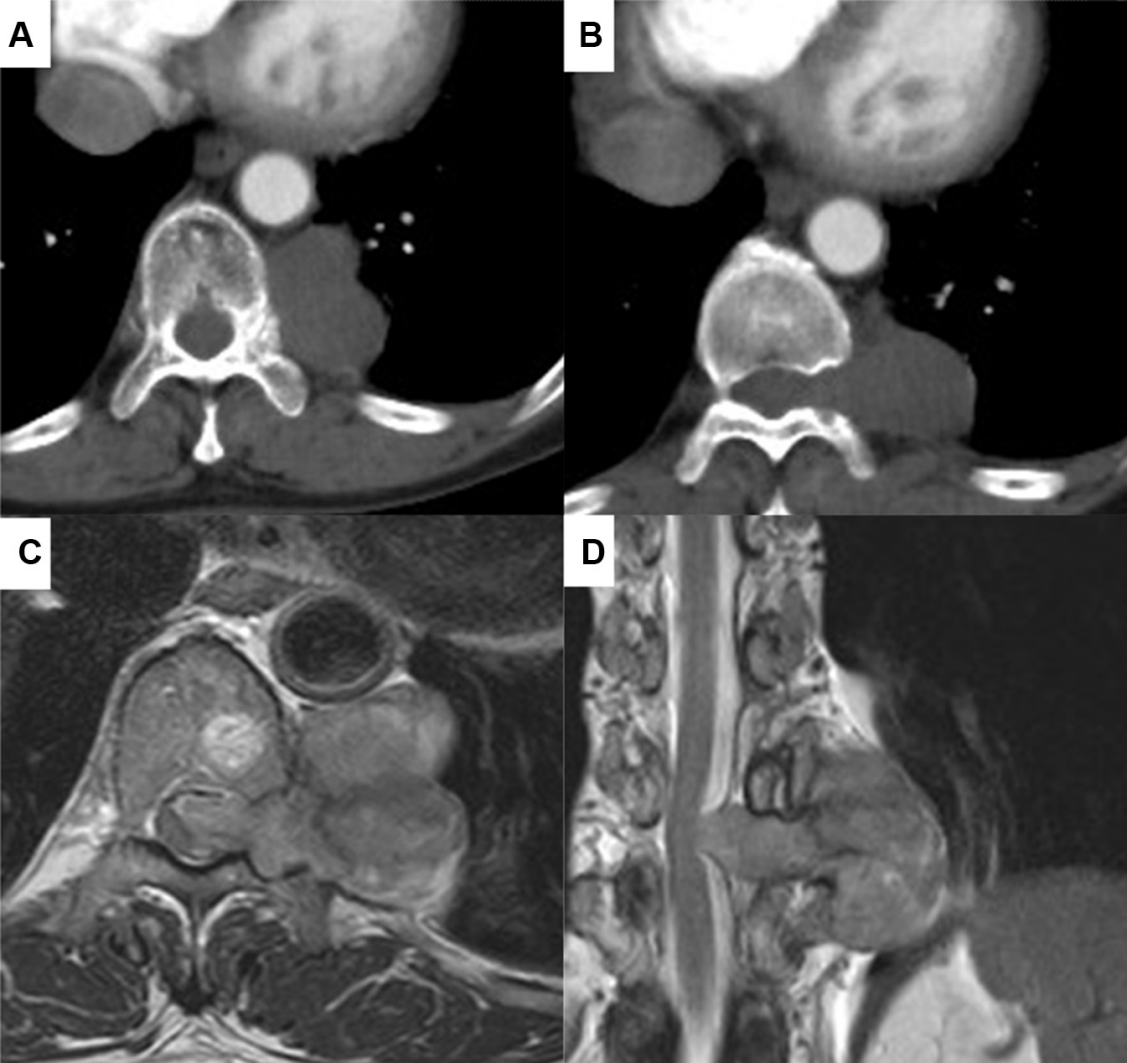
A and B, Computed tomography of the chest images in the axial plane. Magnetic resonance imaging in the axial view (C) and coronal view (D).

The patient, a 65-year-old woman with no remarkable history, was diagnosed with a schwannoma on computed tomography–guided biopsy 13 years ago. The tumor grew gradually and computed tomography of the chest showed a well-defined 4-cm tumor bordering the 10th/11th intercostal space. Part of the tumor extended into the spinal canal, with apparent enlargement of the intervertebral foramen (Figure 1, *A* and *B*). Magnetic resonance imaging showed the tumor extending into the dura mater and compressing the spinal cord from the right side (Figure 1, *C* and *D*).

**Figure 2:**
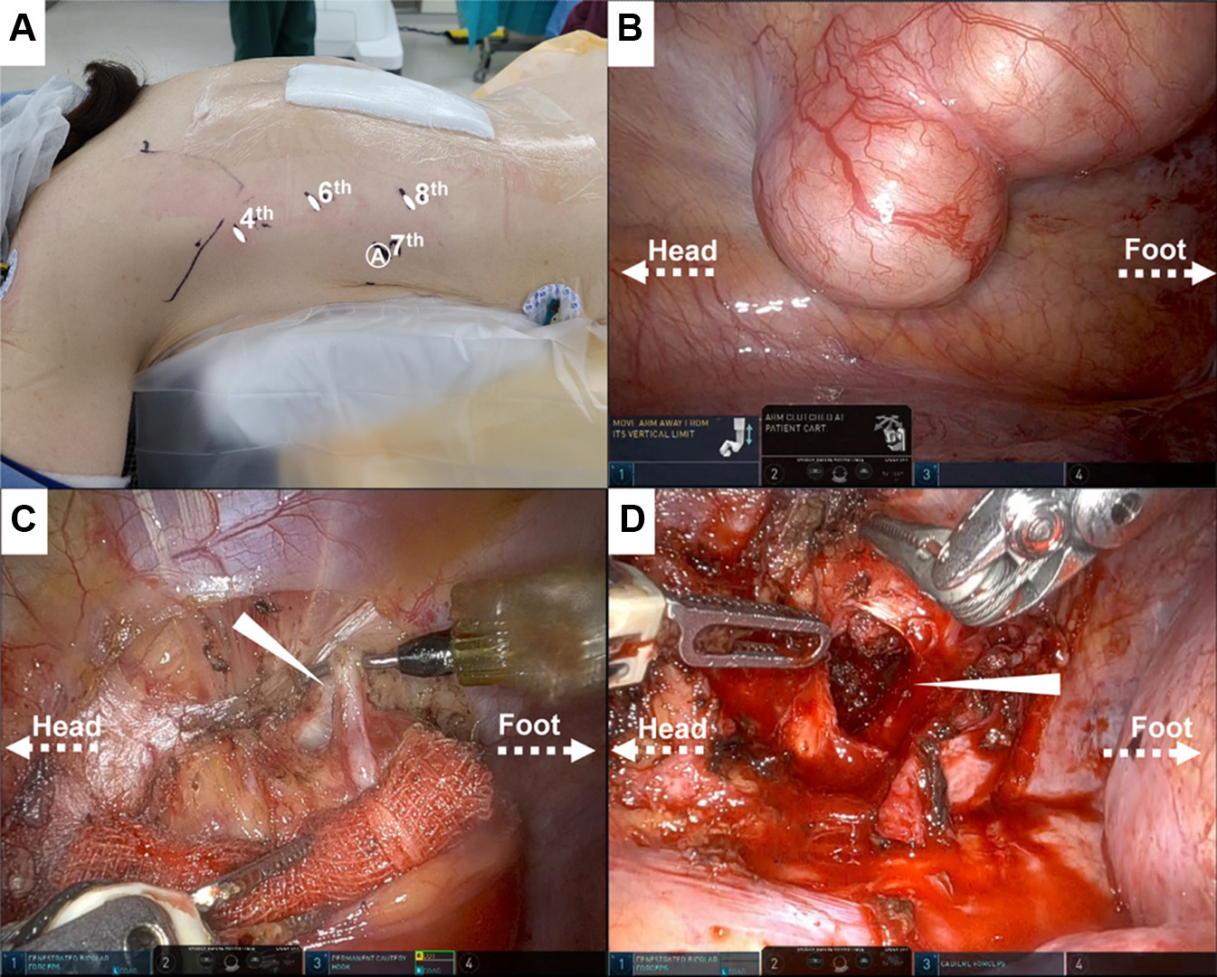
A, Port placement for robotic approach (the A with the *circle* denotes assist), B, intraoperative view of the tumor, C, 10th intercostal nerve (*white arrow*) and D, intraoperative view after tumor resection and intervertebral foramen (*white arrow*).

After general anesthesia was administered, the patient was placed in the prone position (Figure 2, *A*). The dura was incised dorsally to expose the tumor, and the nerve roots contiguous to the tumor were separated within the dura. The tumor was dissected at the level of the intervertebral foramen, and the transverse process and ribs were not resected. The wound was temporarily protected with gauze to terminate the dorsal laminectomy but to allow additional bone resection from the dorsal side if needed for thoracic manipulation. An 8-mm port was placed above the posterior axillary line between the fourth, sixth, and eighth intercostals, and a 12-mm assist port was placed and da Vinci Xi surgical system (Intuitive Surgical) was rolled in, above the midaxillary line in the seventh intercostal space. The fenestrated bipolar forceps was manipulated using the first arm of the robot, and the permanent cautery hook forceps was manipulated by the third arm. The operation up to the visual field and pleural incision around the tumor was performed with CO_2_ insufflated at a pressure of 8 mm Hg. Owing to the dural incision, subsequent operative activities were performed without CO_2_ insufflation to prevent pneumoencephalopathy. The visual field narrowed following CO_2_ insufflation arrest; however, no complications arose during the robotic-assistance phase of the procedure (Figure 2, *B*-*D*, Video 1). The total operative time was 6 hours, 19 minutes, and the console time was 30 minutes. The total blood loss volume was 135 mL, with minimal loss from the thoracic part (5 mL). The tumor was resected in 2 pieces, 1 piece amputated via laminectomy and 1 piece via robotic thoracic approach. After tumor resection, the intervertebral foramen was double-sealed from the dorsal and thoracic sides with fibrin glue and polyglycolic acid sheets to prevent cerebrospinal fluid leak. The surgery was completed without complications and did not require additional dorsal bone resection. The postoperative course was uneventful, with no adverse neurologic symptoms. The excised specimen was histologically diagnosed as a schwannoma.

## Comments

There were various challenges regarding safety. First, there is less surgical field space in RATS, and this increases the likelihood of interference between forceps. The port was placed so that the view was slightly looking down from the head to the caudal side to maintain the distance from the tumor and to accommodate the diaphragmic elevation following the interruption of CO_2_ insufflation; this allowed for a clearer operative view. Despite a narrowed surgical field after CO_2_ insufflation was interrupted, we were able to comfortably manipulate the forceps. Second, the dura mater was opened for back manipulation, and CO_2_ insufflation created a risk of pneumoencephalopathy. Therefore, the back wound was not closed, and the robot was rolled in with only gauze protection. Furthermore, CO_2_ insufflation was performed only until the time of field of view and pleural incision. Third, a discussion on whether dorsal or thoracic manipulation should be performed first was necessary. Dorsal manipulation was performed first because dissecting the central part of the tumor first would prevent potential paralysis or neurological damage caused by unintentional traction by the robot or energization by the electrocautery. In fact, there were no postoperative complications. Finally, because the dorsal wound was kept open due to the risk of pneumoencephalopathy, it was decided to not resect the ribs or transverse process during the dorsal manipulation, and instead add them dorsally as needed in parallel with the thoracic manipulation. McKenna and colleagues^3^ were the first to report the use of VATS to treat posterior mediastinal dumbbell-shaped tumors with the patient in the prone position. However, this method is not widely used due to the technical difficulty of VATS in the prone position. Interestingly, the flexible forceps motion possible through RATS easily dissected the tumor, without additional bone resection. the success of this case exemplifies the feasibility and practical application of this approach.

## Supplementary Data

Video 1 Intrathoracic video of the robot-assisted resection of a dumbbell-shaped tumor via a posterior approach with the patient in the prone position. Video available at: https://www.jtcvs.org/article/S2666-2507(22)00369-8/fulltext.

## References

[1] Li X.K., Cong Z.Z., Xu Y., Zhou H., Wu W.-J., Wang G.-M. (2020). Clinical efficacy of robot-assisted thoracoscopic surgery for posterior mediastinal neurogenic tumors. J Thorac Dis.

[2] Okazaki M., Takigawa T., Suzawa K., Toyooka S. (2021). Robot-assisted intrathoracic procedure for dumbbell tumour in the prone position. Interact Cardiovasc Thorac Surg.

[3] McKenna R.J., Maline D., Pratt G. (1995). VATS resection of a mediastinal neurogenic dumbbell tumor. Surg Laparosc Endosc.

