# Supplementary figures and images for: Robot-assisted resection of a dumbbell-shaped intradural tumor in the prone position
Source: JTCVS Tech. 2022 Jul 2;15:171–3. doi: 10.1016/j.xjtc.2022.06.015 (PMC9579487; doi:10.1016/j.xjtc.2022.06.015)

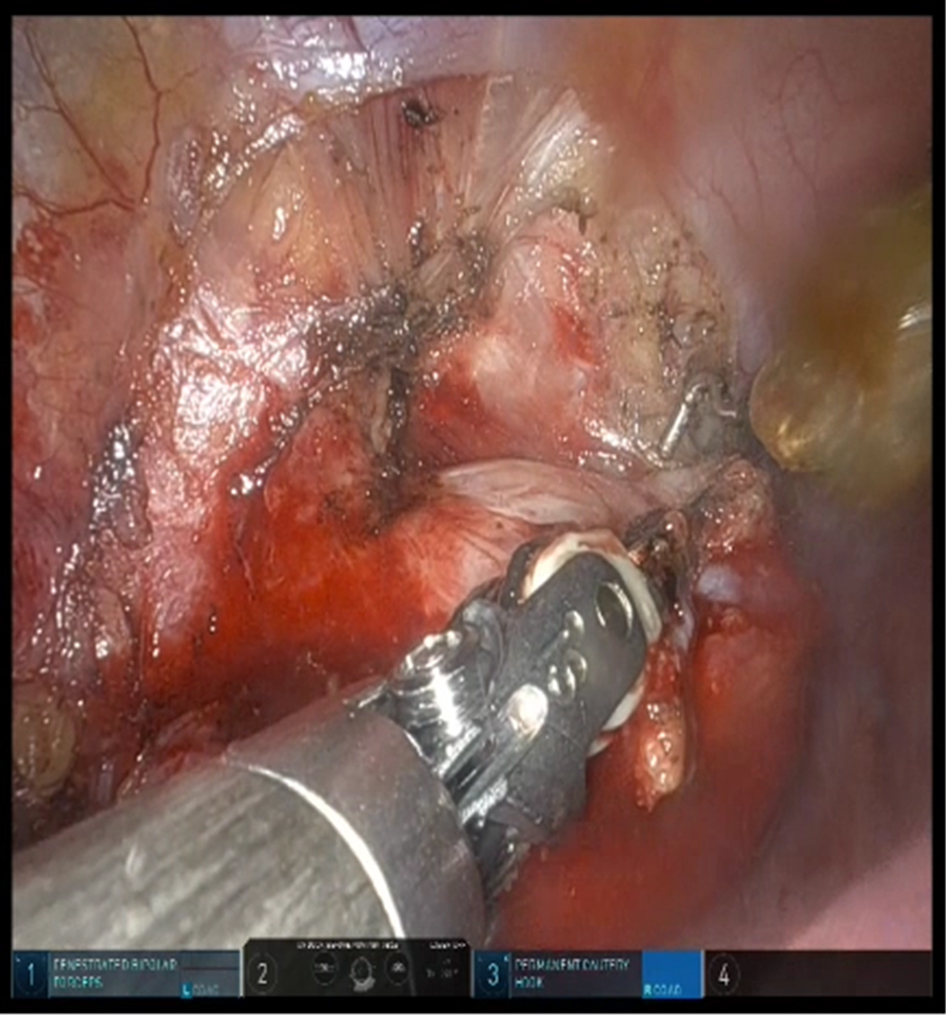

Supplement: Video 1 — Intrathoracic video of the robot-assisted resection of a dumbbell-shaped tumor via a posterior approach with the patient in the prone position. Video available at: https://www.jtcvs.org/article/S2666-2507(22)00369-8/fulltext. [file fx2.jpg]
